# Case Report: Beyond the initial diagnosis: a case of Wilson's disease presenting as acute decompensated cirrhosis

**DOI:** 10.3389/fmed.2026.1973024

**Published:** 2026-10-14

**Authors:** Aun Ali Bangash, Muhammad Ali Bangash, Chloe Cheree Harris, Veronica O’Brien, Learsi De Hoyos, Jose Campo Maldonado

**Affiliations:** 1 Department of Internal Medicine, The University of Texas Rio Grande Valley School of Medicine, Edinburg, TX, United States; 2 Department of Internal Medicine, The University of Texas Health Science Center at San Antonio Joe R and Teresa Lozano Long School of Medicine, San Antonio, TX, United States; 3 Valley Baptist Medical Center – Department of Internal Medicine, The University of Texas Rio Grande Valley School of Medicine, Harlingen, TX, United States

**Keywords:** acute hepatic decompensation, alcoholic hepatitis, copper metabolism, diagnostic challenge, liver transplant, Wilson's diease

## Abstract

Wilson disease is a rare inherited disorder of copper metabolism that can cause progressive hepatic injury, cirrhosis, and, in severe cases, acute liver failure. Its variable presentation can overlap with more common causes of liver disease and delay recognition. We present a 28-year-old patient with one week of worsening jaundice, right upper quadrant abdominal pain, nausea and vomiting, and marked hyperbilirubinemia. Given a history of heavy alcohol use and a Maddrey Discriminant Function score of 73, severe alcohol-associated hepatitis was initially suspected. However, several months of reported alcohol abstinence, a strong maternal family history of early-onset cirrhosis, imaging evidence of established portal hypertension, and the severity of hepatic dysfunction prompted broader evaluation. Testing demonstrated low ceruloplasmin and elevated 24-h urinary copper, while autoimmune and viral testing was unrevealing. The overall clinical evaluation favored Wilson disease. During the hospitalization, suspected hepatorenal syndrome was managed with albumin, midodrine, and octreotide; portal-hypertensive ascites required paracentesis; and trientine plus zinc were prescribed as disease-specific therapy while transplant evaluation was pursued. Renal function improved, but severe liver disease persisted, and the patient was transferred to a transplant center for further evaluation. Long-term follow-up after transfer was unavailable. This case highlights the need to maintain a broad differential diagnosis in young adults with acute decompensation of chronic liver disease and to distinguish clinical diagnostic confidence from the degree of objective Wilson disease confirmation achieved during the index hospitalization.

## Introduction

Wilson's disease is a rare autosomal recessive disorder of copper metabolism caused by variable pathological mutations in the ATP7B gene, which, when unable to maintain copper homeostasis, results in impaired biliary copper excretion and subsequent copper accumulation in the liver, brain, cornea, and other tissues. Although the approximate prevalence is 1 in 30,000 individuals, its clinical symptomatology and presentation are highly variable, ranging from asymptomatic elevation in liver enzymes to more severe presentations such as chronic liver disease, neuropsychiatric sequelae, ophthalmologic findings such as Kayser–Fleischer rings, and fulminant liver failure (1–3). Early recognition is essential as treatment, prognosis, and the potential necessity of liver transplantation all depend on when the patient seeks medical care, severity at presentation, and duration of disease. Furthermore, although patient history may not change the outcome or course of progression of this disease, it is equally important to obtain a thorough patient history to rule out other illnesses that could present similarly to Wilson's disease.

Hepatic involvement is the most common and initial manifestation of Wilson's disease, especially in children and young adults. Patients may show signs of chronic hepatitis, compensated or decompensated cirrhosis, or acute liver failure, often without the simultaneous presence of neurological, psychiatric, or other extrahepatic findings. In addition, acute presentations with the combination of laboratory findings, all but not limited to elevated liver function enzymes, bilirubin, urinary copper excretion, and low levels of ceruloplasmin and serum copper levels, should immediately warrant the consideration of Wilson's disease as a differential, particularly when other etiologies of liver injury are not supported by clinical evidence (1, 4).

One of the greatest diagnostic challenges poses itself when Wilson's disease mimics more prevalent liver disorders, such as alcohol-associated hepatitis and biliary diseases. As hepatobiliary disorders can present with right upper quadrant pain, jaundice, and increased serum bilirubin similar to hepatic involvement in Wilson's disease, patients may undergo hepatobiliary imaging, such as a right upper quadrant ultrasound or hepatobiliary iminodiacetic acid (HIDA) scan, to exclude biliary pathology before less common metabolic disorders are considered. On the other hand, although a patient could have underlying Wilson's disease, their history that indicates the use of excessive alcohol can create a foundation for anchoring bias, leading clinicians to attribute the severe multiorgan clinical symptoms to alcohol while overlooking an inherited metabolic disorder, which in itself can be catastrophic due to the time-dependent variability and quickness of progression (5). Therefore, since Wilson's disease and mimicking conditions look similar but differ substantially, failure to detect it will delay disease-modifying therapy, including timely referral for liver transplantation if acute hepatic decompensation or failure transpires (3, 6).

In this report, we present the case of a 28-year-old male who developed acute hepatic decompensation initially attributed to severe alcohol-associated hepatitis. As the evaluation progressed, the reported period of alcohol abstinence, a strong maternal family history of unexplained early-onset cirrhosis, imaging evidence of established portal hypertension, and abnormal copper studies raised concern for Wilson disease. The case highlights clinical clues that should prompt a structured evaluation for an inherited metabolic liver disorder while avoiding premature diagnostic closure.

## Case presentation

A 28-year-old male with no significant documented past medical history presented with one week of worsening jaundice, right upper quadrant abdominal pain, nausea and vomiting, and diarrhea. He reported heavy alcohol use (3–4 23.5-oz beverages containing 12%–14% alcohol by volume daily for approximately one year) but stated that he had stopped drinking several months before admission. Family history was notable for early-onset unexplained cirrhosis in multiple maternal relatives, raising concern for an inherited liver disorder. On examination, he was alert and oriented, with diffuse jaundice, scleral icterus, right upper quadrant tenderness, ascites, and bilateral lower-extremity pitting edema; serial bedside neurologic examinations documented no focal deficits. Initial and early-admission laboratory studies, summarized in Table 1, demonstrated marked hyperbilirubinemia, coagulopathy, leukocytosis, acute kidney injury, hyponatremia, and hypoalbuminemia. The Maddrey Discriminant Function (MDF) was 73 in the context of the initial working diagnosis of alcohol-associated hepatitis, and the Model for End-Stage Liver Disease (MELD) score was 40. Prednisolone was specifically withheld because the etiology remained uncertain.

**Table 1:** Key admission and early-hospital laboratory findings relevant to the diagnostic and prognostic assessment.

Lab	Result	Reference range	Clinical relevance to Wilson disease
White blood cell count (×10^9^/L)	22.9	4.0–11.0	Marked leukocytosis; contributes to the New Wilson Index.
Hemoglobin (g/dL)	13.6	13.5–17.5 (adult male)	Normal at presentation; hemoglobin subsequently declined during hospitalization. A complete hemolysis evaluation was not documented.
Mean corpuscular volume (fL)	104.5	80–100	Macrocytosis, later attributed clinically to liver disease/folate deficiency rather than proven copper-mediated hemolysis.
Platelet count (×10^9^/L)	257	150–400	Normal at presentation; thrombocytopenia developed later in the setting of advanced liver disease/portal hypertension.
Sodium (mmol/L)	123	136–146	Hyponatremia associated with decompensated cirrhosis.
Blood urea nitrogen (mg/dL)	71	7–18	Marked azotemia accompanying acute kidney injury.
Creatinine (mg/dL)	4.44	0.74–1.35	Acute kidney injury; treated as suspected hepatorenal syndrome.
Albumin (g/dL)	2.6	3.5–5.0	Early-admission hypoalbuminemia; contributes to the New Wilson Index.
Total bilirubin (mg/dL)	>30	0.2–1.2	Marked hyperbilirubinemia in severe hepatic decompensation.
Direct bilirubin (mg/dL)	>10	0.0–0.3	Conjugated hyperbilirubinemia.
Alkaline phosphatase (U/L)	126	laboratory-specific	Admission ALP; later fell to 70 U/L while total bilirubin remained >30 mg/dL.
Aspartate aminotransferase (U/L)	133	10–40	Hepatocellular injury; contributes to Wilson prognostic scoring.
Alanine aminotransferase (U/L)	86	7–56	Hepatocellular injury.
International normalized ratio (INR)	2.05	0.8–1.2	Impaired hepatic synthetic function; contributes to Wilson prognostic scoring.
Maddrey Discriminant Function	73	<32	MDF >=32 supports severe alcohol-associated hepatitis when that diagnosis is present; it is not Wilson-specific.
Model for End-Stage Liver Disease (MELD) score	40	<10	Indicates severe liver dysfunction and supported urgent transplant evaluation.

Given the history of heavy alcohol use and elevated MDF, severe alcohol-associated hepatitis was initially considered. Because the clinical course and family history raised concern for an alternative or additional etiology, further diagnostic evaluation was pursued. CT of the abdomen (Figure 1, left) demonstrated cirrhotic liver morphology with small-volume perihepatic ascites. Noncontrast MRI (Figure 1, right) demonstrated splenomegaly, upper abdominal varices, and moderate ascites, supporting established portal hypertension and cirrhosis without focal hepatic lesions or biliary dilatation. HIDA imaging demonstrated gallbladder filling without cystic duct obstruction or acute cholecystitis; the reported gallbladder ejection fraction was 27%. Together, these studies reduced concern for a focal hepatic lesion or mechanical biliary obstruction as the explanation for the presentation.

**Figure 1:**
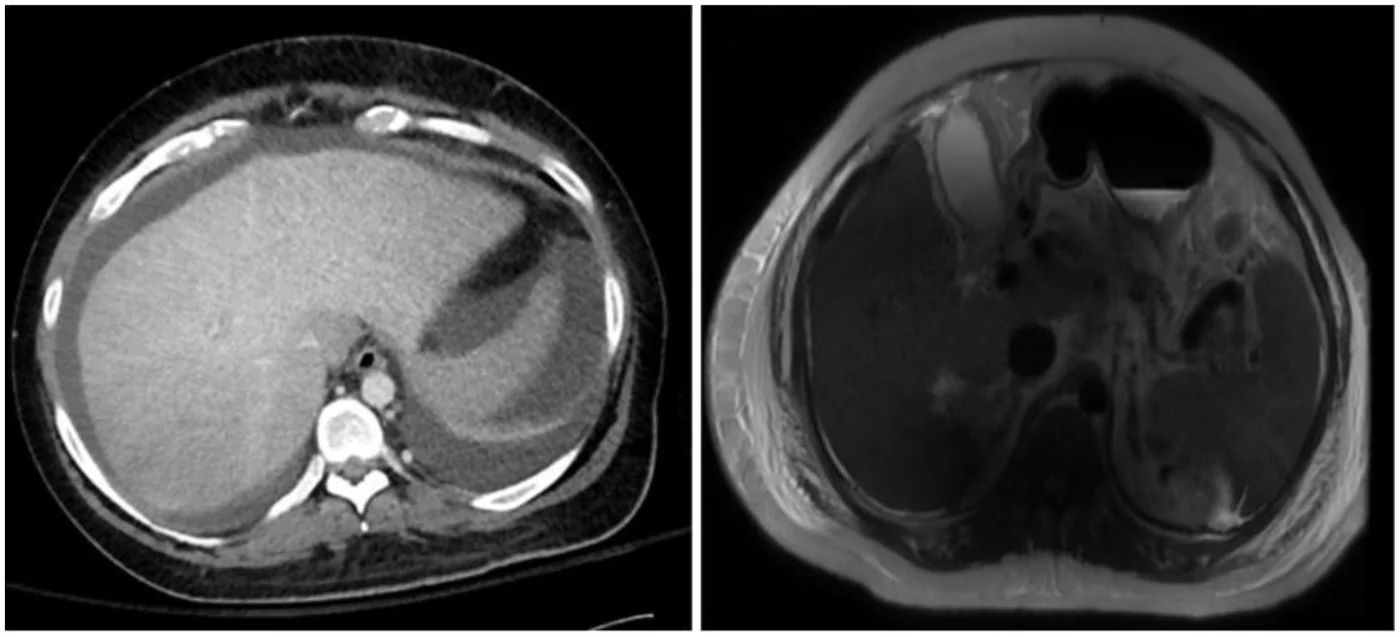
Contrast-enhanced axial CT of the abdomen (left) demonstrates cirrhotic liver morphology with perihepatic ascites. Axial noncontrast MRI of the abdomen (right) demonstrates splenomegaly, upper abdominal varices, and moderate ascites, consistent with portal hypertension secondary to cirrhosis. No focal hepatic lesions or biliary ductal dilatation were identified, and the gallbladder appeared unremarkable.

The evaluation then focused on inherited, autoimmune, and infectious causes of liver disease. Copper studies, autoimmune markers, and viral serologies are summarized in Table 2. Acute viral hepatitis testing was negative by early hospitalization, and autoimmune studies were unrevealing. Low ceruloplasmin and elevated 24-h urinary copper increased suspicion for Wilson disease (13). After multidisciplinary evaluation, including hematology input, the overall presentation was considered most consistent with Wilson disease; however, confirmatory testing remained incomplete before transfer.

**Table 2:** Copper studies, autoimmune markers, and viral serologies including measured results, corresponding reference ranges, and their clinical relevance to Wilson disease.

Lab	Result	Reference Range	Clinical Relevance to Wilson's Disease
Ceruloplasmin (mg/dL)	13.8	20–35	Reduced copper incorporation into ceruloplasmin due to ATP7B dysfunction
Total serum Copper (ug/dL)	60	70–140	Low total serum copper; this finding can occur in Wilson disease because most circulating copper is ceruloplasmin-bound, but serum copper alone is not diagnostic.
24-Hour Urine Copper Excretion (ug/24hr)	131	Previously reported ULN <40 microg/24 h	Elevated 24-hour urinary copper (>2 times the stated upper limit of normal); contributes 2 points to the Leipzig score.
Antinuclear Antibody	Negative	Negative	Autoimmune Hepatitis less likely
Anti-Mitochondrial Antibody	Negative	Negative	Primary Biliary Cholangitis less likely
Smooth Muscle Antibody	Negative	Negative	Autoimmune Hepatitis less likely
Alpha1-Antitrypsin DNA Genetic Test	Negative	Negative	Alpha1-Antitrypsin Deficiency less likely
Anti-Hepatitis A IgM Antibody	Nonreactive	Nonreactive	No serologic evidence of acute hepatitis A
Anti-Hepatitis B Core IgM Antibody	Nonreactive	Nonreactive	No serologic evidence of acute hepatitis B
Hepatitis B Surface Antigen (HBsAg)	Nonreactive	Nonreactive	No serologic evidence of active hepatitis B
Anti-Hepatitis C Antibody	Nonreactive	Nonreactive	No serologic evidence of hepatitis C infection
HIV 1/2 Antibody	Negative	Negative	HIV-associated liver disease less likely
Mononucleosis Heterophile Antibody	Negative	Negative	Acute Epstein–Barr virus infection less likely

Based on the documented copper studies, the Leipzig score was 3 points: ceruloplasmin 13.8 mg/dL (1 point) and 24-h urinary copper 131 microg/24 h, more than twice the laboratory upper limit of normal of 40 microg/24 h (2 points). Under the Leipzig scoring system, a score of 3 indicates that Wilson disease is possible and that additional testing is needed, whereas a score of 4 or greater establishes the diagnosis (14, 15). A slit-lamp examination for Kayser–Fleischer rings was not performed, ATP7B genetic testing was not obtained, and quantitative hepatic copper was not available. A complete evaluation for Coombs-negative hemolysis was also not documented. Given the severity of hepatic decompensation, MELD score of 40, and need for timely evaluation at a transplant-capable center, stabilization and transfer were prioritized and these additional confirmatory studies were not completed locally before transfer.

The patient received supportive management with intravenous albumin, midodrine, octreotide, vitamin supplementation, fluid restriction, and other measures directed at decompensated cirrhosis and suspected hepatorenal syndrome. Portal-hypertensive ascites was confirmed by paracentesis (serum-ascites albumin gradient 2.8), and two therapeutic/diagnostic paracenteses were performed during the hospitalization; ascitic fluid culture was negative. Renal function improved substantially, with creatinine decreasing from 4.44 mg/dL at presentation to 1.92 mg/dL near transfer, although severe liver disease persisted. After multidisciplinary discussion, trientine and zinc were prescribed as Wilson-specific therapy and were included among the transfer medications (trientine 750 mg orally twice daily and zinc sulfate 220 mg orally three times daily). Because of the severity of hepatic decompensation and the need for definitive transplant evaluation, efforts focused on stabilization and securing transfer rather than delaying transfer to complete every confirmatory Wilson study locally. The transplant-referral process extended over the hospitalization while receiving-center acceptance was secured and transplant-related discussions were completed. The patient was ultimately accepted and transferred to a transplant-capable center for formal evaluation approximately 2.5 weeks after admission. The subsequent transplant-center assessment, transplantation status, and longer-term clinical outcome are unknown.

Using the admission and early-admission values, the modified Nazer score was 8 (total bilirubin >30 mg/dL = 4 points; AST 133 U/L = 1 point; INR 2.05 = 3 points), exceeding the historical threshold of 7 associated with poor survival without transplantation. The MELD score was 40. The New Wilson Index (NWI) was 14: bilirubin >30 mg/dL = 4 points, INR 2.05 = 3 points, AST 133 U/L = 1 point, WBC 22.9 × 10^9^/L = 4 points, and early-admission albumin 2.6 g/dL = 2 points. An NWI of 11 or greater has been associated with a high probability of death without liver transplantation (7, 8). Because this patient already had imaging evidence of cirrhosis and portal hypertension, the score is presented as prognostic context rather than as proof of a purely *de novo* acute-liver-failure phenotype.

## Discussion

Wilson's disease is inherited in an autosomal recessive fashion and is caused by various pathogenic mutations in the ATP7B gene. More than 900 mutant variants of this gene have been identified, with the largest proportion being missense mutations, although frameshift, splice-site, and small insertion and deletion mutations have also been identified. The gene itself encodes a copper-transporting ATPase enzyme that is key for binding copper to ceruloplasmin, the body's main copper-transporting protein in the bloodstream, and excreting excess copper into the biliary system within bile. Once dietary copper is absorbed from the duodenum of the small bowel, it enters the portal circulation and is subsequently carried into the intracellular hepatocytes, where it is loaded onto ceruloplasmin and transported to various tissues. As the intracellular concentration of copper rises, ATP7B undergoes a conformational change, allowing itself to be transferred onto the canalicular membrane. This process utilizes ATP hydrolysis and active transport to facilitate copper movement into the biliary system, comprising the body's main excretion mechanism of copper. Therefore, with the loss of ATP7B function, copper builds up in the liver and results in hepatocyte toxicity.

The resultant build-up of free copper within hepatocytes generates reactive oxygen species that cause oxidative damage, mitochondrial dysfunction, lipid peroxidation, and DNA damage, ultimately causing hepatocyte apoptosis and necrosis. Further injury activates hepatic stellate cells, leading to fibrosis and, eventually, cirrhosis and portal hypertension. As liver inflammation progresses, synthetic function declines, resulting in coagulopathy, hypoalbuminemia, hyperbilirubinemia, and acute liver failure in severe cases. Approximately 40%–50% of patients with Wilson's disease initially present with hepatic involvement, whereas fulminant acute liver failure accounts for a small fraction of 5%–8% of presentations but carries a mortality that exceeds 90% without liver transplantation (1, 5). In terminal stages of disease, copper storage becomes overwhelmed and, subsequently, free copper is released into the circulation, accumulates in extrahepatic tissues, and contributes to the multisystem manifestations of advanced Wilson's disease (9, 10).

In this patient, splenomegaly, upper abdominal varices, ascites, cirrhotic morphology, and a portal-hypertensive serum-ascites albumin gradient of 2.8 indicate pre-existing chronic liver disease with portal hypertension rather than a purely *de novo* acute hepatic injury. Thrombocytopenia developed during the hospitalization. The presentation is therefore best framed as acute decompensation of chronic cirrhosis and may represent an acute-on-chronic liver failure phenotype. Formal ACLF classification, however, requires definition-specific organ-failure criteria that are not fully reconstructed in this report. Recognition of these chronic findings in a young adult should prompt consideration of metabolic or genetic liver disease even when a competing exposure, such as alcohol, is present.

The extrahepatic manifestations of Wilson disease are widespread and reflect organ systems susceptible to copper deposition, as summarized in Figure 2. Neurological involvement may include tremor, dystonia, dysarthria, parkinsonism, and psychiatric manifestations. Ophthalmologic findings include Kayser–Fleischer rings and, less commonly, sunflower cataracts. Kayser–Fleischer rings are more frequent in neurologic disease than in isolated hepatic presentations (1, 11). Copper-related disease can also involve the kidneys, heart, and musculoskeletal system (11, 12). In this patient, the clinical manifestations were predominantly hepatic. Serial bedside examinations described the patient as alert and oriented with intact cranial nerves, normal sensation, and no focal neurologic deficits. A formal neurologic consultation was not performed. A slit-lamp examination for Kayser–Fleischer rings was not performed during the index hospitalization and was recommended for follow-up.

**Figure 2:**
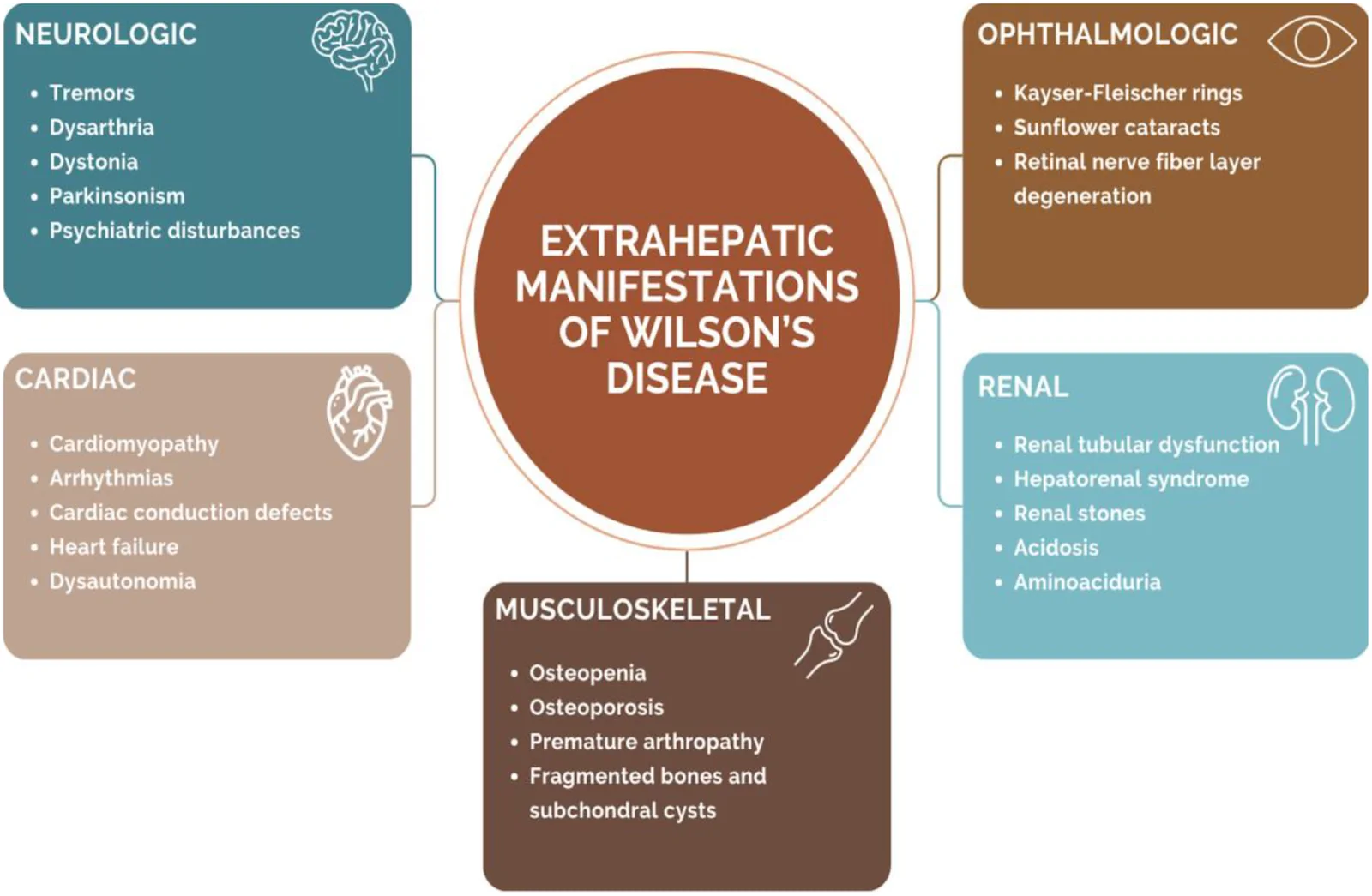
General schematic summary of reported extrahepatic manifestations of Wilson disease. This educational diagram is not intended to represent patient-specific findings in this case.

Treatment of Wilson disease aims to reduce toxic copper burden. Chelating agents such as D-penicillamine and trientine increase urinary copper excretion, while zinc decreases intestinal copper absorption. Current EASL-ERN guidance recommends chelation for significant liver disease; in Wilson disease-related acute liver failure, urgent transplant evaluation remains central, and chelation together with high-volume plasma exchange may be considered as bridging therapy in selected patients (14–17). Recent reports by Delle Cave and colleagues emphasize that intensive disease-specific and supportive therapy may permit native-liver survival in selected cases, while liver transplantation remains lifesaving for patients with severe or progressive failure (16, 17). In this case, trientine and zinc were prescribed before transfer after discussion with hematology and transplant hepatology and were listed among the transfer medications. Plasma exchange was not documented. The patient was ultimately transferred to a transplant center for formal evaluation; the post-transfer treatment course and outcome remain unknown.

## Limitations

This case report is limited by incomplete diagnostic confirmation and lack of post-transfer follow-up. A slit-lamp examination for Kayser–Fleischer rings was not performed, ATP7B genetic testing was not obtained, quantitative hepatic copper was unavailable, and a complete evaluation for Coombs-negative hemolysis was not documented. These studies were not all completed locally because the immediate clinical priority in the setting of severe hepatic decompensation and a MELD score of 40 was stabilization and timely transfer to a transplant-capable center for definitive evaluation. Serial bedside neurologic examinations were nonfocal, but no formal neurologic consultation was performed. Although trientine and zinc were prescribed and the patient was transferred for transplant evaluation, the subsequent transplant-center assessment, transplantation status, and longer-term clinical outcome are unknown.

## Conclusion

Wilson disease should remain in the differential diagnosis of severe liver dysfunction in young adults even when a more common etiology appears plausible. This case illustrates the value of integrating age, family history, signs of chronic portal hypertension, biochemical testing, longitudinal laboratory patterns, and standardized diagnostic and prognostic tools while avoiding anchoring on alcohol exposure alone. When Wilson disease is suspected in a patient with severe hepatic decompensation, disease-specific therapy and early referral to a transplant-capable center should be considered promptly while definitive evaluation proceeds.

## Data Availability

The original contributions presented in the study are included in the article/Supplementary Material, further inquiries can be directed to the corresponding author.

## References

[1] Członkowska A Litwin T Dusek P Ferenci P Lutsenko S Medici V. Wilson disease. Nat Rev Dis Primers. (2018) 4(1):21. 10.1038/s41572-018-0018-3 PMC6416051 30190489

[2] Roberts EA Schilsky ML. Diagnosis and treatment of Wilson disease: an update†. Hepatology. (2008) 47:2089–111. 10.1002/hep.22261 18506894

[3] European Association for Study of Liver. EASL clinical practice guidelines: Wilson’s disease. J Hepatol. (2012) 56(3):671–85. 10.1016/j.jhep.2011.11.007 22340672

[4] Ferenci P. Diagnosis and current therapy of Wilson’s disease. Aliment Pharmacol Ther. (2004) 19:157–65. 10.1046/j.1365-2036.2003.01813.x 14723607

[5] Ala A Walker AP Ashkan K Dooley JS Schilsky ML. Wilson’s disease. Lancet. (2007) 369(9559):397–408. 10.1016/S0140-6736(07)60196-2 17276780

[6] Weiss KH Stremmel W. Clinical considerations for an effective medical therapy in Wilson’s disease. Ann N Y Acad Sci. (2014) 1315:81–5. 10.1111/nyas.12437 24754532

[7] Schilsky ML Roberts EA Bronstein JM Dhawan A Hamilton JP Rivard AM. A multidisciplinary approach to the diagnosis and management of Wilson disease: 2022 practice guidance on Wilson disease from the American association for the study of liver diseases. Hepatology. (2025) 82(3):E41–90. 10.1002/hep.32801 36151586

[8] European Association for the Study of the Liver. EASL-ERN clinical practice guidelines on Wilson’s disease. J Hepatol. (2025) 82(4):690–728. 10.1016/j.jhep.2024.11.007 40089450

[9] Dev S Kruse RL Hamilton JP Lutsenko S. Wilson disease: update on pathophysiology and treatment. Front Cell Dev Biol. (2022) 10:871877. 10.3389/fcell.2022.871877 35586338 PMC9108485

[10] Kasztelan-Szczerbinska B Cichoz-Lach H. Wilson’s disease: an update on the diagnostic workup and management. J Clin Med. (2021) 10:5097. 10.3390/jcm10215097 34768617 PMC8584493

[11] Mulligan C Bronstein JM. Wilson disease: an overview and approach to management. Neurol Clin. (2020) 38(2):417–32. 10.1016/j.ncl.2020.01.005 32279718

[12] Chanpong A Dhawan A. Wilson disease in children and young adults—state of the art. Saudi J Gastroenterol. (2022) 28(1):21–31. 10.4103/sjg.sjg_501_21 35042319 PMC8919932

[13] Delle Cave V Di Dato F Iorio R. Wilson’s disease with acute hepatic onset: how to diagnose and treat it. Children (Basel). (2024) 11(1):68. 10.3390/children11010068 38255382 PMC10814100

[14] Delle Cave V Di Dato F Calvo PL Spagnuolo MI Iorio R. Successful treatment of acute liver failure due to Wilson’s disease: serendipity or fortuity? World J Hepatol. (2024) 16(8):1111–9. 10.4254/wjh.v16.i8.1111 39221095 PMC11362907

[15] Dzieżyc-Jaworska K Litwin T Członkowska A. Clinical manifestations of Wilson disease in organs other than the liver and brain. Ann Transl Med. (2019) 7(Suppl 2):S62. 10.21037/atm.2019.03.30 PMC6531658 31179299

[16] Socha P Członkowska A Jańczyk W Litwin T. Wilson’s disease-management and long term outcomes. Best Pract Res Clin Gastroenterol. (2022) 56−57:101768. 10.1016/j.bpg.2021.101768 35331405

[17] Salman HM Amin M Syed J Sarfraz Z Sarfraz A Sarfraz M. Biochemical testing for the diagnosis of Wilson’s disease: a systematic review. J Clin Lab Anal. (2022) 36(2):e24191. 10.1002/jcla.24191 34951059 PMC8842170

